# Multi-Omics Analysis Reveals Crucial Mechanisms by Which Shading Intensity Regulates Sugar Metabolism in Asparagus Stems

**DOI:** 10.3390/plants15060874

**Published:** 2026-03-12

**Authors:** Qiuxia Li, Gongkai Qiu, Xiaohan Lu, Zhiyuan Liu, Xinyu Zhou, Hu Wang, Fenfen Luo, Mengyao Li, Wei Lu, Chengyao Jiang, Yangxia Zheng

**Affiliations:** College of Horticulture, Sichuan Agricultural University, Chengdu 611130, China; 2025105014@stu.sicau.edu.cn (Q.L.); qiugongkai@stu.sicau.edu.cn (G.Q.); luxiaohan@stu.sicau.edu.cn (X.L.); liu867495948@163.com (Z.L.); zm20030112@163.com (X.Z.); 18509374735@163.com (H.W.); 18381706742@163.com (F.L.); limy@sicau.edu.cn (M.L.); weilu@cau.edu.cn (W.L.); catherinejiang@126.com (C.J.)

**Keywords:** asparagus, metabolome, shade, sugar metabolism, transcriptome

## Abstract

Shade stress is a crucial constraint on asparagus growth in intercropping and dense-planting systems. However, the physiological and molecular mechanisms linking shading intensity to sugar metabolism remain insufficiently understood. Herein, integrating newly generated physiological data with a targeted re-analysis of previously published omics datasets, we elucidated sugar metabolism responses in asparagus stems under different shading intensities (0%, 35%, 55%, and 75%). Moderate shading (55%) was associated with higher sucrose and fructose contents, together with increased activities of key sucrose metabolism enzymes, including sucrose synthase (SUS), soluble acid invertase (S-AI), and sucrose phosphate synthase (SPS), accompanied by differential changes in antioxidant enzyme activities (SOD, CAT and POD). Metabolomic analysis revealed a shift in carbon allocation under 55% shading, characterized by the accumulation of nucleotide sugars such as UDP-galactose and GDP-L-fucose. Transcriptomic analysis further indicated the enrichment of glycolysis/gluconeogenesis pathways under this shading condition, along with the upregulation of pyruvate decarboxylase (PDC) and alcohol dehydrogenase (ADH) genes. Collectively, rather than merely confirming known shading responses, these findings provide new empirical evidence that asparagus stems actively reprogram their energy homeostasis and invoke alternative carbon partitioning pathways specifically at a 55% shading threshold.

## 1. Introduction

Asparagus (*Asparagus officinalis* L.) is a high-value vegetable crop cultivated worldwide. It contains diverse phytochemicals (e.g., flavonoids and saponins) as well as ubiquitous structural carbohydrates like polysaccharides [1] and exhibits antioxidant [2], anticancer [3], antitumor [4], and hypoglycemic [5] effects. Its edibility and market value primarily depend on its tender, succulent stems [6,7]. Unlike grain crops where yield is strictly based on dry biomass, the commercial quality and sensory perception (e.g., Brix and sweetness) of asparagus stems are directly determined by soluble sugar partitioning. Despite the importance of sugars in determining asparagus quality, current studies on the effects of shading on sugar metabolism in asparagus remain extremely limited.

In commercial asparagus production, intercropping with tall-canopy crops (e.g., maize) frequently creates light-reduction environments. These agronomically relevant shading levels typically range from light protective netting to typical intercropping canopies to severe canopy closure [8,9]. In asparagus production systems using dense planting or intercropping, altered light availability is a recurrent management condition that can reshape carbon allocation and stem quality-related metabolism [10,11]. Sugar metabolism connects photosynthetic carbon input with growth, development, and stress responses [12,13]. Studies postulate that low light significantly regulates the activity and gene expression of vital sugar metabolism enzymes, such as sucrose synthase SUS and sucrose phosphate synthase SPS, in crops, including melon and Zhebeimu [14,15,16,17,18,19]. In asparagus, changes in sugar metabolism occur during stems elongation [20], providing a crucial background for studying shade effects. However, despite the importance of sugar metabolism for asparagus stem quality and physiology, the effects of graded shading on asparagus stem sugar metabolism remain insufficiently understood, especially when considered across physiological, metabolomic, and transcriptomic levels.

While our previous broad-scope omics study investigated secondary metabolism and lignin biosynthesis for stem mechanical strength, the reprogramming of primary carbon metabolism remains unexplored. Therefore, we performed a de novo targeted pathway analysis using these raw transcriptomic and metabolomic datasets, rigorously cross-validated by newly quantified sugar fractions and enzyme activities from the exact same biological pools. We hypothesized that varying shading intensities induce directional, non-monotonic alterations in sugar metabolism within asparagus stems that are governed by a critical light intensity threshold. The findings of this study provide new insights into the molecular physiological mechanisms underlying asparagus adaptation to shading stress and offer theoretical support for optimizing field cultivation practices and improving asparagus quality.

## 2. Results

### 2.1. Effects of Different Shade Levels on Sugar Metabolism and Activities of Crucial Enzymes in Asparagus Stems

**Figure 1:**
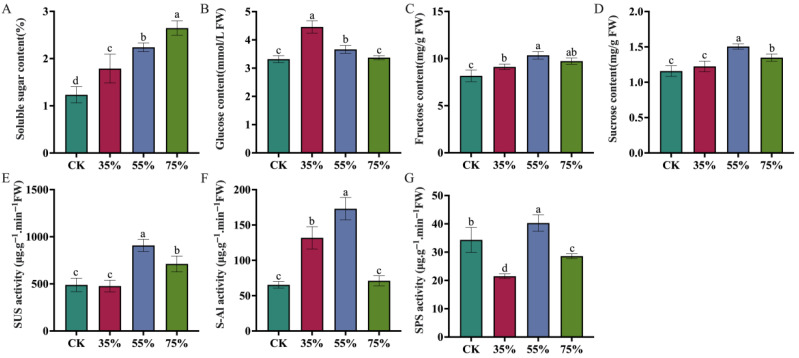
Graphs showing the effects of different shade intensities on the soluble sugar content and the activity of crucial sucrose metabolism enzymes in asparagus stems. (**A**) Soluble sugar content. (**B**) Glucose content. (**C**) Fructose content. (**D**) Sucrose content. (**E**) Sucrose synthase (SUS) activity. (**F**) Soluble acid invertase (S-AI) activity. (**G**) Sucrose phosphate synthase (SPS) activity. CK, 35%, 55%, and 75% represent no shading (control), 35%, 55%, and 75% shading treatments, respectively. Different lowercase letters indicate significant differences between treatments at *p* < 0.05 according to Duncan’s multiple range test.

Different shade treatments (CK, 35%, 55%, and 75%) significantly regulated sugar metabolism and the activities of crucial enzymes in asparagus stems, displaying a distinctly non-monotonic trend across the ordered shading levels. The soluble sugar content exhibited an upward trend with increasing shading intensity (Figure 1A). The glucose content peaked at 35% shading (Figure 1B). In contrast, fructose and sucrose reached their highest levels under 55% shading (Figure 1C,D), indicating specific responses of different sugar components to shading. Of note, sugar components in Figure 1 are presented in specific concentration units relative to fresh weight, all physiological comparisons are standardized and internally consistent across the shading gradients. Sucrose synthase (SUS), soluble acid invertase (S-AI), and sucrose phosphate synthase (SPS) exhibited maximum activity at 55% shading, and subsequently decreased under severe 75% shading (Figure 1E–G). Taken together, different shading intensities exerted distinct effects on sugar composition and the activities of sugar metabolism-related enzymes in asparagus stems. Notably, sucrose content and the activities of sucrose synthesis-associated enzymes were relatively higher under 55% shading, indicating that this shading level may be conducive to the coordinated regulation of sugar metabolism.

### 2.2. Effects of Different Shading Treatments on Growth and Quality-Related Traits of Asparagus Stems

**Figure 2:**
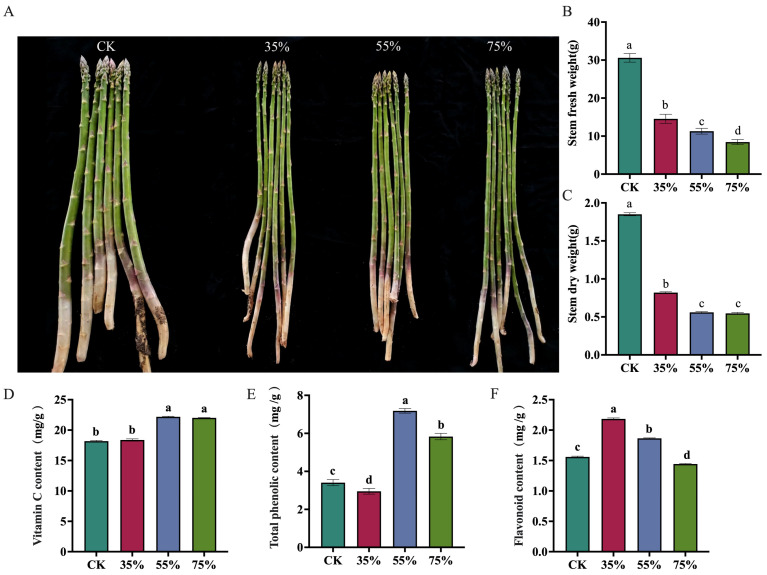
**Effects of Different Shading Treatments on Growth and Quality-Related Traits of Asparagus Stems.** Phenotypic characteristics of asparagus tender stems under different shading treatments (**A**), Fresh weight (**B**), dry weight (**C**), vitamin C content (**D**), total phenol content (**E**), and flavonoid content (**F**) of asparagus stems under different shading treatments, including CK, 35%, 55%, and 75% shading. Different lowercase letters indicate significant differences between treatments at *p* < 0.05 according to Duncan’s multiple range test.

Different shading treatments significantly affected the biomass and quality-related traits of asparagus stems (Figure 2). As shown in the phenotypic observations, increasing shading intensity visibly restricted the overall growth of asparagus (Figure 2A). Both fresh weight and dry weight were highest under CK, and all shading treatments significantly reduced these two parameters. Specifically, the fresh weight exhibited a continuous significant decline as shading intensity increased, while the dry weight also decreased significantly (Figure 2B,C). The vitamin C content was significantly increased under 55% and 75% shading, whereas total phenol content reached its highest level under 55% shading. In contrast, flavonoid content peaked under 35% shading and declined with further increases in shading intensity (Figure 2D–F). Overall, the 55% shading treatment promoted the accumulation of some quality-related compounds (such as vitamin C and total phenols) despite the inevitable reduction in stem biomass.

### 2.3. Effects of Different Shading Levels on Antioxidant Enzyme Activities in Asparagus Stems

**Figure 3:**
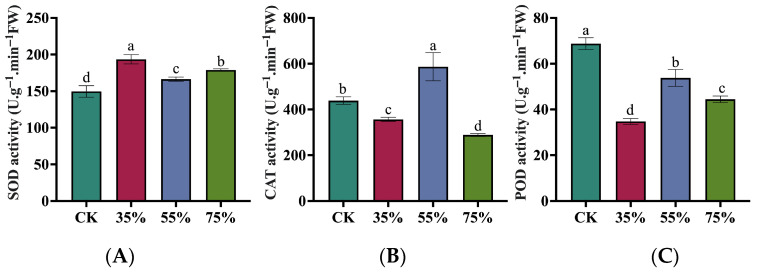
Graphs showing differential responses of asparagus stems to antioxidant enzyme activities under different shade intensities. (**A**) Superoxide Dismutase (SOD) activity. (**B**) Catalase (CAT) activity. (**C**) Peroxidase (POD) activity. Different lowercase letters indicate significant differences between treatments at *p* < 0.05 according to Duncan’s multiple range test.

Different shading levels significantly regulated the antioxidant enzyme system in asparagus stems. Notably, superoxide dismutase (SOD), catalase (CAT), and peroxidase (POD) activities exhibited differential, non-uniform response patterns. SOD activity increased significantly under all shading treatments compared to the control. It exhibited an overall trend of initial increase, followed by a subsequent decrease, and then a slight rise, with the peak occurring at 35% shading (Figure 3A). CAT activity was only significantly higher than the control under 55% shading, but was lower than the control under 35% and 75% shading (Figure 3B). Conversely, POD activity was significantly lower under all shading treatments compared to the control (Figure 3C). Generally, CAT activity was relatively higher under 55% shading, whereas SOD and POD displayed differential responses across shading intensities. These results indicate that rather than a fully coordinated defense, the antioxidant system experiences partial inhibition (as seen in POD) under shading. However, the specific peak of CAT at 55% shading suggests this moderate level may still engage selective mechanisms to alleviate localized oxidative pressure.

### 2.4. Non-Targeted Metabolomics Analysis of Sugar Metabolites in Asparagus Stems Under Shading Treatments

**Figure 4:**
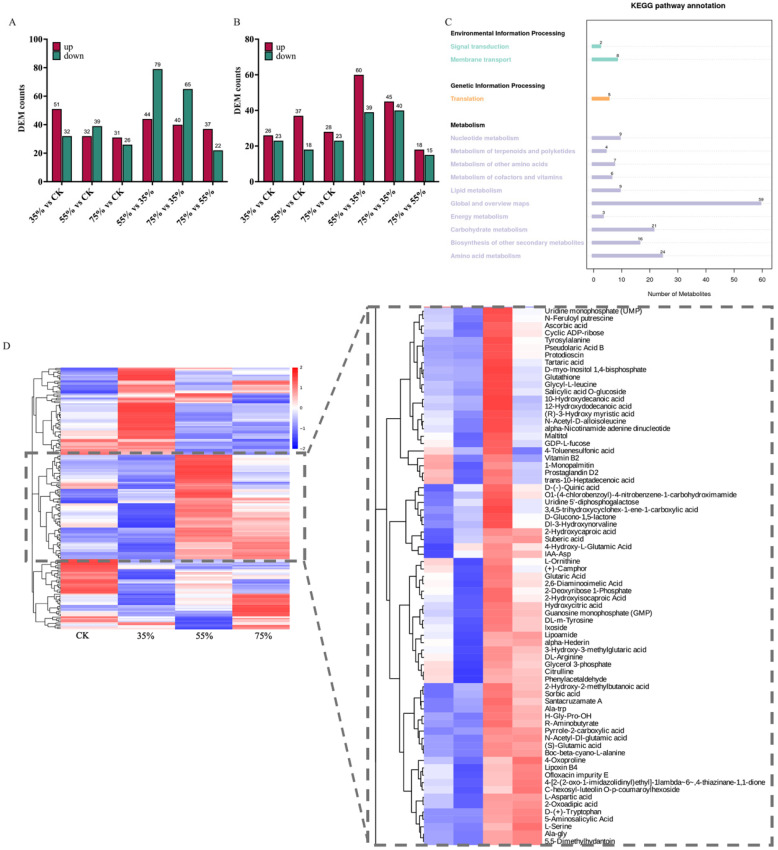
Non-targeted metabolomics analysis results revealing changes in sugar metabolism in asparagus stems induced by shading treatment. (**A**,**B**) Graphs showing the differentially expressed metabolites (DEMs) in up- and downregulated groups under positive (**A**) and negative (**B**) ion modes. (**C**) KEGG enrichment analysis of DEMs in the negative ion mode. (**D**) Clustering heatmap of sugar metabolites. The vital clusters have been highlighted.

This study systematically compared the metabolome profiles across four shading gradients (A (CK), B (35%), C (55%), D (75%)) using the LC-MS/MS technology based on non-targeted metabolomics analysis of sugar metabolites in asparagus stems under shading treatments. Differential metabolite (DEM) screening revealed distinct responses to shading across positive and negative ion modes. The number of up-regulated DEMs exceeded that of the down-regulated DEMs in the 35% vs. CK, 75% vs. CK, and 75% vs. 55% groups, while the opposite trend was observed in the 55% vs. CK, 55% vs. 35%, and 75% vs. 35% groups in the positive ion mode (Figure 4A). In contrast, all comparison groups in the negative ion mode were dominated by upregulated DEMs (Figure 4B). The KEGG pathway metabolites differentially detected in the negative ion mode were further annotated to focus on the sugar metabolism network, given that sugar compounds are efficiently detected primarily via this mode. This phenomenon revealed significant enrichment in global and overview maps, as well as in amino acid and carbohydrate metabolism (Figure 4C). Hierarchical clustering analysis further revealed the predominant clustering of sugar metabolites, including derivatives such as maltitol, GDP-L-fucose, uridine 5′-diphosphogalactose (UDP-galactose), and D-glucono-1,5-lactone, within the gray dashed box (Figure 4D). Although distinguishing sugar nucleotide isomers in non-targeted LC-MS/MS can be challenging without authentic standards, the MS/MS fragmentation patterns and exact mass database matching provided Metabolomics Standards Initiative (MSI) Level 2 confidence for these annotations. The accumulation patterns of these metabolites indicated that shading stress triggers sugar metabolism pathways in asparagus stems, particularly through adaptive changes in negative ion-sensitive metabolites, such as sugar nucleotides and sugar derivatives, in response to light energy deficiency.

### 2.5. Transcriptome Analysis of Sugar Metabolism in Asparagus Stems Under Shade Treatment

**Figure 5:**
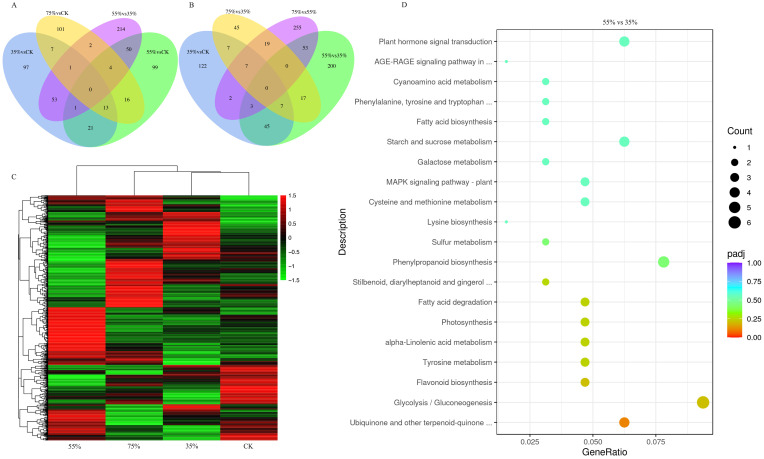
Transcriptomic analysis results revealing crucial shifts in sugar metabolism in asparagus stems under 55% shading. (**A**,**B**) Venn diagrams comparing differentially expressed genes (DEGs) among treatments with varying shading intensities. (**C**) Hierarchical clustering analysis results of the union of DEGs across all comparison groups. (**D**) KEGG pathway enrichment analysis results of DEGs in the 55% vs. 35% comparison group. The glycolysis/gluconeogenesis pathway (ko00010) was significantly enriched.

RNA-seq analysis was performed on samples from the four shading gradients to elucidate the transcriptional regulatory mechanisms governing sugar metabolism in asparagus stems under shading stress. High-quality transcriptome data were obtained after rigorous quality control [21]. Venn diagram analysis of differentially expressed genes (DEGs) revealed that the 55% vs. 35% and 75% vs. 55% groups contained over 200 unique DEGs, whereas the 75% vs. 35% group contained only 45 unique DEGs (Figure 5A,B). This non-monotonic shift further supports that the 55% shading intensity acts as a critical physiological inflection point. DEGs in the 55% vs. 35% group were the only significantly enriched in the core pathway of sugar metabolism, i.e., glycolysis/gluconeogenesis (ko00010) (Figure 5D), based on clustering analysis of the union of DEGs across six comparison groups (Figure 5C) and KEGG enrichment results. Notably, marker genes indicative of severe metabolic shifts among the DEGs mapped to the significantly enriched glycolysis/gluconeogenesis pathway, two alcohol dehydrogenase genes (LOC109830273 and LOC109823954) and three pyruvate decarboxylase genes (LOC109843566, LOC109855578, and LOC109840037) showed prominent induction patterns, and were therefore highlighted as representative responsive genes under 55% shading (Supplementary Table S1).

### 2.6. Integrating Transcriptome and Metabolome Analysis of Sugar Metabolic Pathways in Asparagus Stems Under Shade Treatment

**Figure 6:**
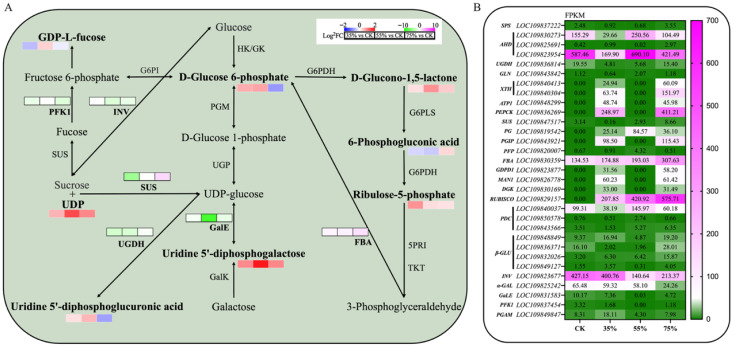
Integrated metabolomic and transcriptomic analysis results of sugar metabolism in asparagus stems under shading stress. (**A**) Flow diagram showing the effects of shading intensity on the accumulation of crucial sugar metabolites and gene expression. Differentially expressed metabolites and genes are presented as red–blue and purple–green heatmaps, respectively. Arrows indicate the direction of metabolic flux. (**B**) The expression patterns of crucial sugar metabolism enzyme genes. SPS: sucrose phosphate synthase; AHD: alcohol dehydrogenase; UGDH: UDP-glucose dehydrogenase; GLN: glucan endo-1,3-β-glucosidase; XTH: xyloglucan endo-transglucosylase; ATP1: Phospholipid transporter ATPase 1; PEPCK: Phosphoenolpyruvate carboxykinase; SUS: Sucrose synthase; PG: Polygalacturonase; PGIP: Polygalacturonase inhibitor protein; FBA: Fructose-1,6-bisphosphate aldolase; GDPD1: Glycerophosphodiester phosphodiesterase 1; MAN1: Mannose glycosidase 1; DGK: Diacylglycerol Kinase; RUBISCO: Ribulose Bisphosphate Carboxylase Small Subunit; PDC: Pyruvate Decarboxylase; β-GLU: β-Glucosidase; INV: β-Furanosyl fructosidase; α-GAL: α-Galactosidase; GalE: Galactose epimerase; PFK1: Phosphofructokinase 1; PGAM: 2,3-Diphosphoglycerate-dependent phosphoglycerate mutase.

Integration of transcriptome and metabolome data revealed that shade stress significantly impacts sugar metabolism in asparagus stems. 6-phosphogluconic acid, uridine 5′-diphosphoglucuronic acid, uridine 5′-diphosphogalactose, D-glucono-1,5-lactone, uridine diphosphate (UDP), and GDP-L-fucose were among the metabolites showing relatively higher abundance under 55% shading than in the control group (CK), with the largest fold changes observed in the 55% vs. CK comparison (Figure 6A). Sugar metabolism processes are finely regulated by crucial enzymes at the gene expression level. Galactose epimerase GalE (LOC109831583) was downregulated across the three treatments, with the lowest expression under 55% shading, suggesting its inhibitory role in regulating sugar metabolism. Conversely, sucrose synthase SUS (LOC109847517) was significantly suppressed at 35% shading and upregulated at 75% shading. UDP-glucose dehydrogenase UGDH (LOC109836814), responsible for oxidizing UDP-glucose to UDP-glucuronic acid, exhibited significant suppression at 35% and 55% shading compared to 75% shading. However, fructose bisphosphate aldolase FBA (LOC109830359) exhibited a sustained upregulation with increasing shading intensity, reflecting its continuous responsive role in glycolysis. Notably, the expression levels of crucial enzymes, such as GalE, SUS, and UGDH, remained low across all treatments, potentially because of their catalytic activity or metabolic demand (Figure 6B). These findings indicated that shading stress enhances the stress tolerance of asparagus stems by promoting the synthesis of protective metabolites, such as GDP-L-fucose, through the coordinated regulation of multiple metabolic pathways, including glycolysis, sucrose metabolism, cell wall degradation, and nucleoside synthesis. It should be noted that the accumulation of sugar nucleotides and sugar derivatives primarily reflects the redistribution and regulatory adjustment of sugar metabolic pathways under shading conditions, rather than serving as direct evidence for enhanced sucrose degradation or increased monosaccharide accumulation.

## 3. Discussion

This study systematically explored the response characteristics of sugar metabolism in asparagus stems under different shading intensities by nominal shading-net treatments by integrating physiological/biochemical measurements with targeted re-analysis of previously generated metabolomic and transcriptomic datasets. The results demonstrate a close association between variations in shading intensity and sugar metabolic processes in asparagus stems, with the moderate shading treatment (55%) exhibiting response patterns distinct from those of other treatments across multiple sugar metabolism-related indicators, suggesting that this shading level may represent a relatively coordinated metabolic adaptation and a critical quality-yield trade-off of asparagus under light-limited conditions.

At the physiological level, the sucrose and fructose content, as well as the activity of crucial sucrose metabolism enzymes, including SUS, S-AI, and SPS, reached their relatively highest under moderate 55% shading (Figure 1). The continuous increase in total soluble sugars suggests continuous carbon accumulation, but only under 55% shading did sucrose and fructose reach their peak levels, indicating selective carbon allocation at this level. This supports the idea that 55% shading is a finely regulated inflection point. It is important to acknowledge that increased sugar concentrations under shading can sometimes theoretically arise from a passive “concentration effect” driven by reduced growth. To address this, we evaluated stem biomass (Figure 2A–C). As shading intensity increased, stem biomass exhibited a continuous and significant decline (CK > 35% > 55% > 75%). If the sugar accumulation were solely a passive concentration effect driven by reduced water content or biomass, the 75% treatment (which had the lowest biomass) should have exhibited the highest sugar concentration, which contradicts our findings, since the level of sucrose, the primary product of photosynthetic carbon assimilation—significantly dropped at 75% (Figure 1D). Therefore, coupled with the strong upregulation of active sucrose synthesis enzymes (Figure 1E–G), this confirms an active reconfiguration of sink strength rather than passive dilution. Furthermore, the significant accumulation of health-promoting phytochemicals, including total phenolics and vitamin C, specifically at 55% shading (Figure 2C,D), underscores its functional role in enhancing the comprehensive nutritional and market quality of asparagus stems despite the inevitable yield penalty. This finding suggests that the intermediate shading treatment was associated with altered carbohydrate allocation and sucrose metabolism-related regulation in asparagus stems. Previous studies have shown that shifting carbohydrate resources toward soluble sugars, thereby increasing their accumulation to maintain osmotic balance and provide substrates for metabolism [22]. The results of the present study are consistent with this view. Meanwhile, these non-uniform response patterns indicate that the antioxidant system undergoes a complex asymmetric adjustment rather than a fully coordinated defense. As the first line of defense, SOD activity was significantly induced under all shading conditions (peaking at 35%), indicating a rapid physiological response to convert superoxide radicals into hydrogen peroxide. However, the downstream scavenging enzymes exhibited divergent strategies. While the universal suppression of POD implies a partial impairment of the baseline detoxification capacity, the specific and robust induction of CAT at 55% shading serves as a vital compensatory mechanism. This suggests that at this moderate shading level, asparagus stems selectively shift their reliance toward CAT to efficiently process the hydrogen peroxide generated by SOD, thereby alleviating localized oxidative pressure [23,24]. These results suggest that moderate shading may partially buffer shading-induced oxidative pressure but does not lead to a comprehensive enhancement of the antioxidant system. The parallel modulation of sugar metabolism and antioxidant responses may jointly contribute to the adaptation of asparagus stems to shaded environments. We propose that the 55% threshold represents the limit of phenotypic plasticity; below this level, homeostasis is maintained via shade avoidance, while above it (75%), severe light limitation induces metabolic dormancy.

Non-targeted metabolomics provides intrinsic evidence for the aforementioned phenotypes at the metabolite level. Notably, crucial differentially expressed metabolites, including nucleosides such as UDP-galactose and GDP-L-fucose (Figure 4D), were identified in the negative ion mode, clearly delineating the directional shift in carbohydrate flux under shading stress. These metabolites are not merely energy carriers but also play crucial signaling and structural roles. For instance, GDP-L-fucose often accumulates during cell wall remodeling, thereby enhancing stress signaling through fucosylation of proteins and cell wall polysaccharides, a crucial mechanism for plants to enhance stress tolerance [25,26,27]. Similarly, the accumulation of D-glucaric acid-1,5-lactone potentially indicates the activation of the pentose phosphate pathway, which supplies reducing power (NADPH) for various biosynthetic reactions and produces pentoses for nucleotide synthesis, collectively countering oxidative stress [28,29]. Sugar nucleotides play essential roles in cell wall polysaccharide biosynthesis, protein glycosylation, and stress-related signal transduction. Therefore, the results of this study suggest that, under shading conditions, asparagus stems may support their structural integrity and metabolic adaptation through the modulation of sugar nucleotide metabolism.

Transcriptome analysis revealed the regulatory basis of metabolic changes at the gene expression level. The glycolysis/gluconeogenesis pathway was significantly enriched only in the 55% vs. 35% comparison group (Figure 5D), strongly suggesting that a 55% shading threshold represents a critical point for initiating significant alterations in genes associated with core carbon metabolism. Notably, representative significantly changed genes identified in the glycolysis/gluconeogenesis pathway, particularly pyruvate decarboxylase (PDC) and alcohol dehydrogenase (ADH), were associated with the fermentative branch of carbon metabolism and may reflect a selective energy-maintenance response under moderate shading, are hallmark responses when plants shift to fermentation pathways under carbon starvation or energy limitation to maintain ATP and NAD+ supply [20,21,22,23,24,25,26,27,28,29,30,31,32]. Interestingly, this induction was stronger at moderate (55%) than at severe (75%) shading. We hypothesize that at 55% shading, the stems are still attempting to maintain essential elongation and developmental processes, creating a high localized ATP demand that outpaces aerobic respiration under reduced light. In contrast, extreme 75% shading likely forces the plant into global metabolic suppression and dormancy, downregulating even these emergency pathways. These changes in gene expression may reflect the regulatory demands of asparagus for maintaining energy homeostasis under photosynthetically limited conditions.

Herein, moderate shading (55%) promoted the accumulation of sucrose and fructose in asparagus stems, consistent with the general trend of soluble sugar accumulation in various crops under shading or stress conditions. For instance, the vacuolar acid invertase gene SlVI directly influences fruit quality and postharvest stress tolerance by regulating sugar metabolism in tomatoes [33]. In this study, alterations in SlVI function led to significant changes in sucrose, fructose, and glucose content, thereby affecting fruit flavor quality and stress resistance during storage. Similarly, short-day shading treatment significantly increases soluble sugar content by promoting sucrose redistribution, starch degradation, and enhancing sucrose storage capacity in citrus fruits [34]. Citrus fruits also activate a complex set of molecular and physiological mechanisms during drought stress, thereby promoting the accumulation of soluble sugars [35]. These findings collectively suggest that plants mobilize sugar metabolism to maintain intracellular metabolic equilibrium when facing environmental stress [36].

Asparagus stems also exhibited unique metabolic adaptation mechanisms in response to specific shading intensities. Asparagus significantly enhanced the synthesis of nucleoside sugars, such as UDP-galactose and GDP-fucose, under 55% shading, unlike crops, such as citrus, which primarily accumulate soluble sugars in response to shading. While this profile could partially reflect the specific detection window of our non-targeted metabolomics approach, it strongly indicates that carbon allocation is redirected to support cell wall remodeling. This phenomenon suggests that carbon allocation is directed towards energy storage and osmotic regulation, as well as supporting cell structure and signaling molecule synthesis. This metabolic profile reflects the specialized strategy of asparagus to maintain structural integrity and adapt to stress under low-light conditions, making it a suitable stem vegetable. Moreover, asparagus primarily responds to light limitation through adjustments in endogenous energy metabolism pathways, unlike maize, which relies on exogenous nitrogen fertilizer to promote growth under low light [37]. Genes associated with glycolysis and fermentation, including PDC and ADH, were specifically induced under 55% shading, indicating that asparagus activates alternative energy production pathways to sustain basic energy supply. This mechanism contrasts sharply with the strategies of most field crops under shading, which rely on morphoplasticity or nutrient uptake. Notably, the expression patterns of some vital glycolytic genes did not fully correlate with the accumulation patterns of their respective metabolites, suggesting the presence of potential post-transcriptional regulatory mechanisms. This phenomenon aligns with Hartman et al.’s (2023) perspective that plant carbon metabolism is finely tuned by allosteric effects and post-translational modifications [38], further illustrating the multi-level and multi-factor complexity of shade response regulation in asparagus.

This study further revealed a vital regulatory pathway through transcriptomic-metabolomic correlation analysis (Figure 6). Galactose epimerase (GalE) expression was significantly suppressed under 55% shading, potentially leading to UDP-galactose accumulation and hindering its conversion to UDP-glucose. Notably, fructose-1,6-bisphosphate aldolase (FBA) remained upregulated, providing a stable metabolic foundation for glycolysis. Conversely, the significant upregulation of UDP-glucose dehydrogenase (UGDH) under intense shading (75%) signaled a further metabolic shift, where increased UDP-glucose was directed toward synthesizing UDP-glucuronic acid, a vital precursor for cell wall polysaccharides, such as pectin and hemicellulose [39,40,41]. These phenomena reveal the complexity of multi-level regulation. For instance, UGDH transcripts peaked at 75% shading, but its putative product UDP-glucuronic acid accumulated most abundantly at 55% shading. This phenomenon strongly suggests that efficient regulation of translational or enzymatic activity ensures an adequate supply of cell wall precursors at the critical threshold of 55% shading. Furthermore, discrepancies between transcripts and metabolites were observed. Rather than solely post-transcriptional control, these mismatches likely reflect inherent time-lag effects between rapid gene transcription and slower metabolite accumulation, differential subcellular compartmentation, or post-translational controls, which are common in dynamic stress responses captured at a single time point. Similarly, the discrepancy between gene expression and activity for certain vital enzymes involved in sugar metabolism reinforces our conclusions. It reveals a significant scientific finding that sugar metabolism responses in asparagus under shading stress undergo multi-level, fine-tuned regulation spanning transcription to post-translational modifications. This finding aligns closely with recent perspectives on the complexity of plant metabolic regulations [38].

Nonetheless, future studies should focus on functional validation of the crucial genes identified in this study, including ADH, FBA, and UGDH. Furthermore, while this study relies on manufacturer-rated nominal shading levels to establish physiological thresholds, comparing our findings to other quantitative shading studies highlights a limitation. Future research incorporating continuous measurements of photosynthetic photon flux density (PPFD), daily light integral (DLI), and canopy microclimate alongside net ratings will be invaluable for precisely defining the biophysical drivers of these metabolic shifts. These studies should employ in vivo and in vitro genetic approaches to elucidate their specific roles in shade response. Results from these studies would provide precise molecular targets for breeding more shade-tolerant asparagus varieties, ultimately enhancing their adaptability under dense planting systems or when intercropped with other crops.

## 4. Materials and Methods

### 4.1. Plant Materials and Treatments

Asparagus cultivar ‘Fengdao No. 2’ (Sichuan Jinnong Seed Co., Ltd., Chengdu, China) was used in this study. Four shading gradient treatments were established at a site in Enyang District, Bazhong City, Sichuan Province, China, using asparagus plants that had been planted for three years and had visually uniform growth, with no obvious symptoms of disease or mechanical damage. Plant uniformity was assessed based on overall plant vigor and stem developmental status before treatment establishment.

The treatments included no shading (CK, full light), 35% shading, 55% shading, and 75% shading. Shading was achieved by setting up a steel frame and covering it with black polyethylene shading nets (Taizhou Jinhui Netting Co., Ltd., Taizhou, China) of different shading rates (manufacturer-rated nominal shading levels: different nominal shading percentages). The 35%, 55%, and 75% treatments were selected to represent a gradient from mild to severe light reduction commonly used in horticultural shading experiments and to allow comparison across a broad response range.

Each treatment plot had an area of 60 square meters (5 m × 12 m) and was arranged in a randomized block design with three replicates. The field layout followed a randomized block design with three plot replicates per treatment (one plot per treatment within each block), and the plot was considered the experimental unit for field-level inference. Fifty healthy asparagus stems with a similar growth status (assessed visually based on comparable stem developmental stage and uniform external vigor) and approximately 35 cm in length were randomly selected from each treatment after 21 days of shading for physiological, transcriptomic, and metabolomic sampling. To minimize diurnal bias, sampling was conducted simultaneously across treatments between 10:00 and 11:00 AM. Furthermore, to eliminate positional effects, stems were cut exactly 23 cm from the apex, and only the middle segments were pooled into a single composite sample per plot. Transcriptomic analysis was done in three biological replicates, while non-targeted metabolomic analysis was conducted in six biological replicates. For physiological measurements, statistical analyses were conducted using plot-level biological replicates. All samples were immediately flash-frozen in liquid nitrogen upon collection and stored at −80 °C.

### 4.2. Measurement of Physiological Parameters

The soluble sugar content of asparagus was determined using the anthrone-sulfuric acid colorimetric method following the procedure described by Li et al. [42]. Glucose, fructose, and sucrose contents were measured using the method described by Bouzika et al. [43]. The activities of enzymes associated with sugar metabolism, including SUS, S-AI, and SPS, were measured and quantified following the method described by Yu et al. [44]. Vitamin C content was determined using the xylene extraction colorimetric method [45]. Flavonoid and total phenol contents were determined using the HCl–methanol extraction colorimetric method [45]. Plant biomass was evaluated based on fresh and dry weight measurements. The fresh weight of each stem segment was recorded, and the samples were then placed in paper envelopes and dried in an oven at 65 °C for 72 h until constant weight, after which the dry weight was determined. SOD, POD, and CAT activities were measured following the method described by Xiao et al. [45]. All biochemical reagents were purchased from Chengdu Kelong Chemical Co., Ltd. (Chengdu, China).

### 4.3. Transcriptome and Non-Targeted Metabolome Analysis

The transcriptomic and non-targeted metabolomic analyses of asparagus stems were based on the high-throughput sequencing and metabolite detection data previously completed [21]. With the premise of controlling the quality of the raw data, this study focused on the scientific question of shading intensity and sugar metabolism response and performed re-selection, systematic integration, and targeted analysis of the transcriptomic and metabolomic data.

Transcriptome sequencing was performed on the Illumina HiSeq 4000 (Illumina, Inc., San Diego, CA, USA) platform. Clean reads were aligned using HISAT2 (v2.0.5; Johns Hopkins University, Baltimore, MD, USA), and differentially expressed genes (DEGs) were selected using DESeq2 (v1.20.0; Bioconductor project, Frederick, MD, USA) based on the criteria of |log_2_FC| ≥ 1 and FDR < 0.05. Metabolomic data were obtained using the UHPLC-MS/MS platform (a Vanquish UHPLC (Thermo Fisher Scientific, Waltham, MA, USA) coupled to an Orbitrap Q Exactive HF mass spectrometer (Thermo Fisher Scientific, Waltham, MA, USA) via a Hypersil Gold column (100 *times 2.1 mm, 1.9 **μm**; Thermo Fisher Scientific, Waltham, MA, USA)). Raw data were processed in Compound Discoverer 3.1 for peak alignment, and metabolites were annotated against KEGG, HMDB, and LIPIDMaps databases with MSI Level 2 confidence, and differential metabolites (DEMs) were selected based on the criteria of VIP > 1 and *p* < 0.05.

On this basis, this study focused on the changes in genes and metabolites related to sugar metabolism and, combined with physiological and biochemical indicators, conducted a comprehensive analysis of the sugar metabolism regulation patterns induced by shading.

### 4.4. Statistical Data Analysis

Data were analyzed using IBM SPSS Statistics 26 software and presented as means ± standard deviation. Because preliminary tests showed no significant block effects across the field, data were analyzed using a one-way ANOVA, treating the three biological composite pools (representing the 3 plots) as the independent experimental units. Significant differences between means were assessed using Duncan’s multiple range test. The level of statistical significance was set at *p* < 0.05 (denoted by different lowercase letters). The GraphPad Prism 9.5.1 software was used for creating various data visualization graphs. Each measured parameter had three biological replicates.

## 5. Conclusions

**Figure 7:**
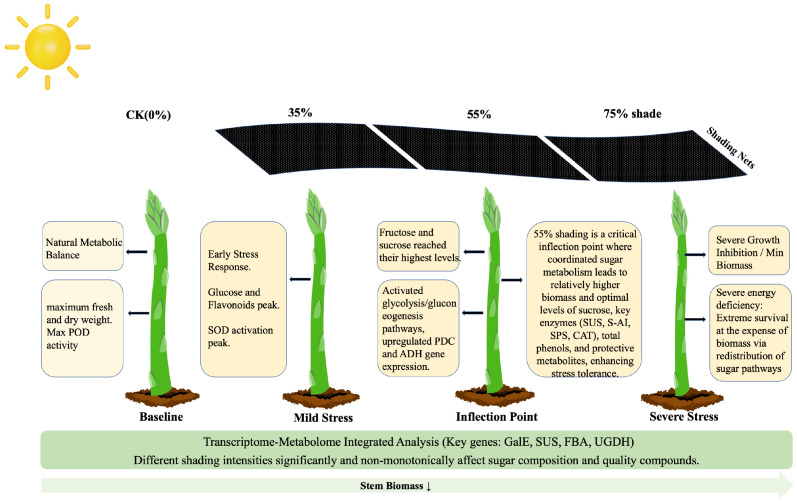
Pattern diagram of asparagus tender stem development under shading conditions. The black arrows point to the summarized physiological and metabolic characteristics.

This study systematically revealed that different shading conditions can significantly influence the sugar metabolism characteristics of asparagus stems (Figure 7). Under a shading intensity of 55%, asparagus stems exhibited relatively coordinated response patterns in terms of sugar component contents, the activities of sucrose metabolism-related enzymes, and the levels of certain sugar metabolism-related genes and metabolites. This was manifested as synergistic increases in the activities of crucial sucrose metabolism enzymes and accumulation of soluble sugars. Integrated transcriptomic and metabolomic analysis revealed a specific shift in carbon allocation towards nucleotide sugar synthesis pathways characterized by significant accumulation of GDP-L-fucose and UDP-galactose, under 55% shading intensity. These metabolic changes were associated with the expression patterns of representative responsive genes, including ADH, PDC, and FBA, which together suggest selective remodeling of carbon metabolism under 55% shading. This discovery provides new evidence for understanding the carbon metabolic plasticity of asparagus. Rather than serving as immediate cultivation guidance, these findings provide fundamental mechanistic insights into energy homeostasis and nutritional quality enhancement. The identified genes serve as hypothesis-generating candidates for future functional validation.

## Data Availability

Data will be made available on request.

## Supplementary Materials

The following supporting information can be downloaded at: https://www.mdpi.com/article/10.3390/plants15060874/s1, Table S1: KEGG Bubble Diagram Pathway Annotations for Each Comparison Group (A = CK, B = 35%, C = 55%, D = 75%).
