# Supplementary material for: Multi-Omics Analysis Reveals Crucial Mechanisms by Which Shading Intensity Regulates Sugar Metabolism in Asparagus Stems
Source: Plants (Basel). 2026 Mar 12;15(6):874. doi: 10.3390/plants15060874 (PMC13030577; doi:10.3390/plants15060874)
Supplement: Supplementary file 1 [file plants-15-00874-s001.zip › plants-4155224-supplementary.pdf]

Table S1: KEGG Bubble Diagram Pathway Annotations for Each Comparison Group （A=CK、B=35%、C=55%、D=75%）

Description (Sorted by *p*-value)	geneName	group
Glycolysis / Gluconeogenesis	LOC109830273/LOC109823954/ LOC109843566/LOC109855578/ LOC109840037	CvsB up
Glycosphingolipid biosynthesis - globo and isoglobo series	LOC109825242	DvsB down
Glycosphingolipid biosynthesis - globo and isoglobo series	LOC109825242	DvsA down
Starch and sucrose metabolism	LOC109836371/LOC109847517	DvsB up
Amino sugar and nucleotide sugar metabolism	LOC109831583/LOC109836814	CvsA down
Starch and sucrose metabolism	LOC109823677/LOC109849127/ LOC109848849	CvsB down
Glycolysis / Gluconeogenesis	LOC109830273/LOC109823954	BvsA down
Starch and sucrose metabolism	LOC109848849/LOC109836371/ LOC109837222	DvsC up
Glycerophospholipid metabolism	LOC109855247/LOC109829265	DvsC up
Glycerophospholipid metabolism	LOC109855247	DvsB up
Pentose and glucuronate interconversions	LOC109836814	CvsA down
Fructose and mannose metabolism	LOC109837454	CvsA down
Glycerophospholipid metabolism	LOC109819611	CvsA up
Glycerophospholipid metabolism	LOC109827356	BvsA down
Amino sugar and nucleotide sugar metabolism	-	BvsA down
Fructose and mannose metabolism	LOC109820007	DvsC down
Glycerophospholipid metabolism	LOC109827356	CvsB up
Starch and sucrose metabolism	-	BvsA down
Amino sugar and nucleotide sugar metabolism	LOC109831583	CvsB down
Glycolysis / Gluconeogenesis	LOC109849847	CvsB down
Starch and sucrose metabolism	LOC109843842	CvsB up
Glycerophospholipid metabolism	LOC109847594	DvsC down
Glycolysis / Gluconeogenesis	LOC109825691	DvsC up
Glycolysis / Gluconeogenesis	LOC109820007	DvsC down
